# Sphingolipids in Non-Alcoholic Fatty Liver Disease and Hepatocellular Carcinoma: Ceramide Turnover

**DOI:** 10.3390/ijms21010040

**Published:** 2019-12-19

**Authors:** Jorge Simon, Alberto Ouro, Lolia Ala-Ibanibo, Natalia Presa, Teresa Cardoso Delgado, María Luz Martínez-Chantar

**Affiliations:** 1 Liver Disease and Liver Metabolism Lab, CIC bioGUNE, Centro de Investigación Biomédica en Red de Enfermedades Hepáticas y Digestivas (CIBERehd), 48160 Derio, Bizkaia, Spain; loliaibanibo@gmail.com (L.A.-I.); tcardoso@cicbiogune.es (T.C.D.); mlmartinez@cicbiogune.es (M.L.M.-C.); 2 Department of Biochemistry and Molecular Biology, Faculty of Science and Technology, University of the Basque Country (UPV/EHU), 48980 Leioa, Bizkaia, Spain; alber.ouro@gmail.com (A.O.); npretor@gmail.com (N.P.); 3 Instituto Biofisika (UPV/EHU, CSIC), University of the Basque Country, 48940 Leioa, Spain

**Keywords:** Metabolic syndrome, NAFLD, NASH, cirrhosis, HCC, sphingolipids, ceramide, S1P, sphingomyelin, metabolomics, lipidomics

## Abstract

Non-alcoholic fatty liver disease (NAFLD) has emerged as one of the main causes of chronic liver disease worldwide. NAFLD comprises a group of conditions characterized by the accumulation of hepatic lipids that can eventually lead to non-alcoholic steatohepatitis (NASH), fibrosis, cirrhosis, and hepatocellular carcinoma (HCC), the fifth most common cancer type with a poor survival rate. In this context, several works have pointed out perturbations in lipid metabolism and, particularly, changes in bioactive sphingolipids, as a hallmark of NAFLD and derived HCC. In the present work, we have reviewed existing literature about sphingolipids and the development of NAFLD and NAFLD-derived HCC. During metabolic syndrome, considered a risk factor for steatosis development, an increase in ceramide and sphigosine-1-phosphate (S1P) have been reported. Likewise, other reports have highlighted that increased sphingomyelin and ceramide content is observed during steatosis and NASH. Ceramide also plays a role in liver fibrosis and cirrhosis, acting synergistically with S1P. Finally, during HCC, metabolic fluxes are redirected to reduce cellular ceramide levels whilst increasing S1P to support tumor growth.

## 1. Non-Alcoholic Fatty Liver Disease and Derived Hepatocellular Carcinoma

**Figure 1:**
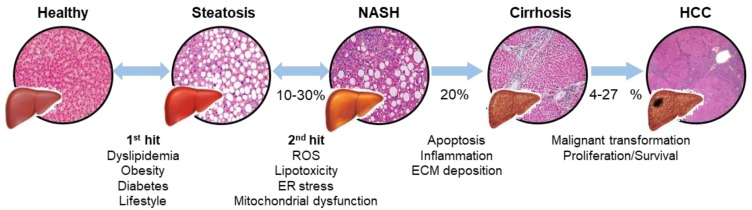
Liver disease progression: from steatosis to hepatocellular carcinoma. Schematic representation of the two-hit hypothesis and the progression of the disease, with characteristic H&E staining micrographs from each state of the pathology. A first hit leads an increased lipid accumulation in the liver, named steatosis. In a *second* hit reactive oxygen species (ROS), lipotoxicity, endoplasmic reticulum (ER) stress and mitochondrial dysfunction lead to non-alcoholic steatohepatitis (NASH) in 10%–30% patients. Sustained damage results in apoptosis, inflammation and extracellular matrix (ECM) deposition, leading to a fibrotic response and cirrhosis in 20% of patients. Finally, the 4%–27% of chronic patients can develop hepatocellular carcinoma (HCC).

NAFLD is a term that encompasses a group of pathologies ranging steatosis, non-alcoholic steatohepatitis (NASH) and cirrhosis (Figure 1). This condition is particularly manifested in Western countries, with an incidence rate of about 20%–30% in the general population [1,2]. The most accepted explanation for NAFLD progression is the two-hit hypothesis [3], where an initial first hit induces lipid accumulation in the liver and increases liver vulnerability to different factors (second hits). As Sanyal notably reviewed, such second hits consist on an increased endoplasmic reticulum (ER) and oxidative stress, followed by a decreased antioxidant capacity that creates a pro-inflammatory environment and contributes to mitochondrial dysfunction [4]. 

On the other hand, lipotoxicity has also been pointed out as a second hit during NAFLD progression [5]. In this context, several works point out the convergence between metabolic pathways from triglycerides and sphingolipids. Indeed, steatosis has been correlated with an increased sphingolipid content, suggesting that lipid imbalance generates bioactive lipids that might act together with other second hits during NAFLD progression [6,7,8]. Steatosis progresses into NASH in 10%–30% cases with subsequent inflammation, hepatocyte death and fibrosis development [9]. Around 20% of NASH patients are estimated to develop cirrhosis if fibrosis development persists [10,11]. Although sometimes a compensated cirrhosis without symptoms can occur, it is often followed by complications leading to liver dysfunction [12].

Furthermore, cirrhosis can also evolve to hepatocellular carcinoma (HCC) with poorer survival rates [13]. HCC is the most prevalent form of presentation (70%–85%) of liver cancer, which is the fifth most common cancer type in the world and the second cause of cancer-related death [14,15]. Around 4%–27% of NAFLD patients are estimated to develop HCC development [16,17], and NAFLD is positioning as one of most leading causes of HCC and the most increasing one [18]. Improvement in understanding and diagnosing, particularly in NAFLD-derived HCC, is mandatory for finding suitable therapies and diagnosing methods against the disease.

## 2. Sphingolipid Metabolism: Ceramide as Central Molecule

**Figure 2:**
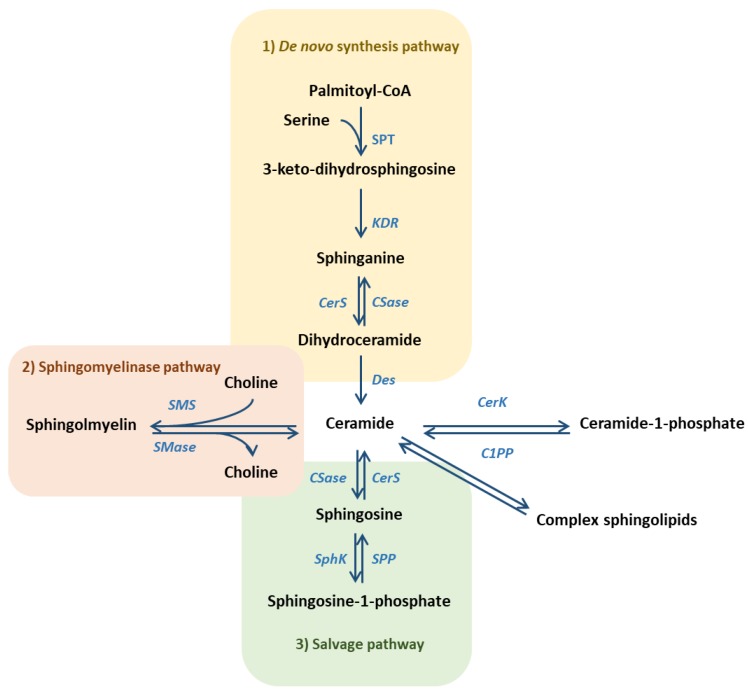
Ceramide is the central molecule in sphingolipid metabolism and can be synthesized by three different pathways: de novo synthesis pathway, sphingomyelinase pathway and salvage pathway. (SPT = serine palmitoyltransferase; KDR = 3-keto-dihydrosphingosine reductase; CerS = ceramide synthase; CSase = ceramidase; Des = desaturase; SphK = sphingosine kinase; SPP = S1P phosphatase; SMase = sphingomyelinase; SMS = sphingomyelin synthase; CerK = ceramide kinase; C1PP = ceramide-1-phosphate phosphatase).

Sphingolipids are bioactive molecules that play a role in several cell functions and participate in tissue homeostasis [19,20,21]. Ceramide is considered as the central molecule in sphingolipid metabolism and it has been reported to play a pro-apoptotic and anti-proliferative role, regulating tissue homeostasis [22,23]. On one hand, ceramide can be synthetized from three different pathways (Figure 2): i) In the de novo pathway, by condensation of serine and palmitate, ii) through sphingomyelin (SM) catabolism in the sphingomyelinase (SMase) pathway, and iii) through the catabolism of complex sphingolipids in the salvage or catabolic pathway. The relationship between ceramide production and inflammation has been highly described by various authors [24,25]. Conversely, ceramide content can be reduced by its conversion into SM or complex glycosphingolipids (GSL), a common mechanism from cancer cells to block ceramide-induced cell death signaling [26]. Otherwise, ceramide can be directly phosphorylated into ceramide-1-phosphate (C1P), which mediates in many inflammatory processes through a specific membrane receptor [27,28,29]. 

*Ceramidase* catabolizes ceramide into sphingosine, which can be further phosphorylated into sphingosine-1-phosphate (S1P) (Figure 2), described as a pro-mitogenic and anti-apoptotic molecule [30]. There are two sphingosine kinase (SphK) isoforms 1 and 2 [31] that, together with phosphatases, control sphingosine/S1P turnover and make them exert intra- and extra-cellular functions [32,33]. Extracellular S1P can act through a specific G-protein coupled receptor family, named S1PR, closely linked with S1P-mediated inflammation [34,35].

Taking into account sphingolipid contribution to cell and tissue homeostasis and metabolism, as well as the combination of perturbations observed during NAFLD and derived HCC development, many studies have pointed out sphingolipids as potential triggers of the disease [25,36].

## 3. Sphingolipid Contribution to Metabolic Syndrome

Pathologies from metabolic syndrome (MetS) such as obesity, dyslipidemia and insulin resistance (IR) have been reported to induce first hits that induce steatosis development [37,38]. Noteworthy, diabetes and overweight increase the prevalence of the pathology from 20%–30% to 30%–50% and 80%–90% respectively, becoming almost universal when both are combined [37]. Metabolic perturbations during MetS may lead to alterations in the homeostasis of sphingolipids.

In this context, ceramide synthase (CerS), involved in ceramide production in the de novo and salvage pathways, has been described to promote weight gain, glucose intolerance [39,40] and the development of IR [41]. Meanwhile, CerS inhibition by fenretinide has also been demonstrated to prevent lipid-induced IR [42]. Moreover, an upregulation in de novo synthesis through serine-palmitoyltransferase (SPT) modulation has been reported to increase ceramide content in serum [43], whereas selective inhibition with myriocin decreases ceramide levels and ameliorates obesity-derived atherosclerosis [44,45], improves insulin sensitivity [40,43] and reduces body weight [46]. Remarkably, in the study performed by Park and colleagues they have observed a myriocin-induced reduction in hepatic de novo lipogenesis (DNL) that ameliorates serum hyperlipidemia and hypercholesterolemia [47]. It has been also reported an insulin sensitivity recovery through SPT inhibition in high-fat diet (HFD)-induced IR mouse models, which also lead to steatohepatitis and NASH [40,48].

Several works have also characterized the contribution of adipose tissue to sphingolipid content, where a fatty acid excess promotes ceramide synthesis through the de novo pathway. Furthermore, obesity-derived processes promote the release of pro-inflammatory cytokines, such as tumor necrosis factor (TNF), that stimulate ceramide synthesis [49,50] and contribute to NAFLD progression [5]. Indeed, reduced ceramide content by myriocin has been reported to promote adipose tissue beiging with a subsequent improvement in insulin sensitivity and resolution of hepatic steatosis [51]. Therefore, an increase of lipid content promotes ceramide synthesis, not only in the liver but also in adipocytes, contributing to a pro-inflammatory environment.

In relation to the mechanisms by which ceramides inhibit insulin signaling, Jebailey et al. indicates the reduction in the translocation of the glucose transporter type 4 (GLUT4) as a key one [52]. Furthermore, it has been also reported that ceramide interacts with protein kinase B (PKB/Akt) preventing its activation by insulin and inhibiting glucokinase activation, which inhibits glucose uptake and its conversion into glycogen [53,54]. Pertaining to Akt signaling, ceramide-induced inhibition has been described to be mediated by protein phosphatase *2* (PP2A) activation [55]. In a work from our laboratory, Zubiete-Franco et al. have already reported the effect of PP2A activation over autophagy dysregulation in the development of steatosis. Therein, an excess of S-adenosylmethionine (SAMe) inactivates PP2A resolving the pathology [56]. In this context, the dysregulation of autophagy caused by an activation of PP2A by ceramide could be another risk factor that, combined with PKB/Akt-mediated IR, contributes to steatosis.

As mentioned above, ceramide can be metabolized to S1P by the action of ceramidase and SphK, described to be increased in serum from obese patients that suggest a role of ceramide in MetS development [57,58]. An increased fatty acid uptake by the hepatocyte has been observed to stimulate C_16_-Ceramide and S1P synthesis with a direct effect on IR development [59,60,61]. Interestingly, the work from Shimabukuro also points a possible role of palmitate on promoting the apoptosis of pancreatic beta-cells [60]. Therefore, the effect of ceramide and S1P synthesis would act synergistically with beta-cells apoptosis and inhibiting downstream signaling for promoting insulin resistance.

Inhibition of SPT and CerS (particularly CerS6) presents an attractive target for avoiding MetS-derived steatosis and NASH development. In fact, type 2 *diabetes mellitus* (T2DM) has been reported to be reverted followed by a reduction on ceramide levels in patients [62] through a treatment that also resolves the NAFLD phenotype [63]. The correlation of MetS, the increase of sphingolipid content (particularly ceramide and S1P) and the development of steatosis and NASH prove to be strongly correlated.

## 4. Ceramide and Other Sphingolipids in Steatosis and NASH

As aforementioned, steatosis is characterized by an abnormal lipid accumulation, which can lead to NASH through the production of second hits [3]. Imbalances in sphingolipid homeostasis and IR are so relevant in NASH development that mice fed the HFD, the most extended model for IR, also develop NASH with a characteristic steatosis, inflammation, and fibrosis [64]. This may be due to the existing link between triglycerides and sphingolipids synthesis [65,66], combined to the fact that liver lipid content, and sphingolipids particularly, is higher than other tissues [67,68].

Several works have reported increased SM levels in liver [69] and serum [44] from HFD-fed mice, while SM reduction ameliorates both insulin sensitivity and steatosis [44,45]. Cano and colleagues have also reported elevated SM levels in methionine adenosyltransferase 1a (Mat1a)-deficient mice, which spontaneously develop NASH through a disrupted hepatic very-low density lipoprotein (VLDL) assembly [70,71]. Relevantly, around 50% of human NASH patients present a serum metabolic profile associated to *Mat1a*^-/-^ mice [72], which suggests that SM might also be upregulated in NASH patients. Regarding another NASH animal model, an elevated sphingolipid content has been also characterized in mice overexpressing *diacylglyceride-transferase 2* (*Dgat2*), where an increased lipogenesis leads to lipid accumulation without IR [73]. 

SMase is a family of enzymes that can be distinguished by their pH optima [74]. In this context, acid SMase (A-SMase) has been reported to be upregulated in NAFLD [6] and its deficiency has been related to the prevention of lipid accumulation in the liver [75]. Likewise, elevated ceramide levels, product of SM catabolism, have been related to the pathology [76,77] as they can promote mitochondrial ROS production by interfering in the electron transport chain, thus leading to an oxidative stress increase [78]. Oxidative stress is one of NASH hallmarks and responsible for inflammation and fibrosis development, so that ceramide could play a role in NAFLD progression by enhancing the effect on other second hits [7]. Moreover, some studies have characterized the relevance of ceramide fatty acid composition in their biological effect, pointing C_16_-ceramides as mediators in NASH [39,79]. In fact, when CerS6, the enzyme responsible of C_14_/C_16_-ceramide synthesis, is downregulated, there is a prevention of steatosis as ceramide also acts as a negative regulator of β-oxidation [39]. Interestingly, the supplementation with Vitamin E also improves NASH development with a subsequent ceramide reduction [71], so that enrichment with this compound in patients offers a compelling therapy.

Inflammation has been widely reported to play a key role during NASH progression into further stages by promoting hepatocyte cell death, Kupffer cell (KC) activation and hepatic stellate cell (HSC)-mediated fibrosis development [5,80]. In this context, cytokines such as TNF and other interleukins are also associated with NAFLD progression from NASH to further stages and, remarkably, they have been reported to upregulate ceramide production [25]. Relating to this, Chang and collaborators have reported that TNF and interleukin-1 (IL-1) activate SPT [81], while inhibiting its effect reduces ceramide content and reverts NASH development [82]. TNF-mediated biological effect has been reported to be through its binding with tumor necrosis factor receptor 1 (TNFR1) and SMases (A-SMase and possibly neutral N-SMase) activation [83], together with the promotion of ROS production [84]. In the meantime SMases also downregulate reduced glutathione (GSH) content in the hepatocyte, reducing the antioxidant capacity of the cell and promoting the effect exerted by ROS excess [85,86]. The treatment with adiponectin, besides improving insulin sensitivity and ameliorating lipid accumulation in the liver, also mitigates the effect of TNF in the hepatocyte while it promotes the anti-inflammatory cytokine IL-10 secretion by KCs [87].

Ceramide has been reported to accumulate in lipid droplets forming acyl-ceramide by the DGAT2-CerS-ACSL5 complex [88]. Considering the pro-apoptotic effect of the sphingolipid in the hepatocyte [89], this could explain the ceramide accumulation observed in steatosis and initial stages of NASH with absent cell death. However, at later stages, ceramide accumulation could be so high that the complex DGAT2-CerS-ACSL5 would be unable to conjugate all ceramide, so that the molecule would be present in its free form triggering hepatocyte cell death and leading to fibrosis development [88,89,90]. It would be interesting to perform an exhaustive study in order to determine whether the ceramide effect in each stage of the pathology depends on its conjugation into lipid droplets and if this process mediates in NAFLD progression.

Current routine methods are a key challenge for accurate correct NASH diagnose, and research is focused on finding adequate markers that allow to identify the pathology in a cost-effective way [1,91]. In this context, the lipidomic profile characterization of serum from NASH patients has identified an upregulation of ceramide levels [39,58]. Correlated to this, Alonso and another authors have also based on metabolomics analysis of serum to point ceramide and SM as key promoters of the transition between steatosis and NASH [72,77]. Therefore, ceramides may present a suitable method to identify the development and progression of NAFLD.

In summary, the high-fat hepatic content during MetS might lead to the observed hepatic SM accumulation. Meanwhile, enhanced SM catabolism through A-SMase upregulation could lead to the observed ceramide increase in NASH. Regarding NASH lean patients, more research needs to be performed for characterizing sphingolipid contribution to the pathology, as ceramide could be somehow mediating in the development of hepatic IR.

## 5. Ceramide and S1P Role in Fibrosis and Cirrhosis Development

During fibrosis development, hepatocyte cell death activate KCs that release pro-inflammatory cytokines, activating HSCs [80,92]. Once activated, HSCs secrete extracellular matrix (ECM) that fills the space of Disse and proliferate, replacing dead hepatocytes by fibrotic scar tissue which turns into fibrosis under chronic deposition [90,93,94].

Regarding their effect in the hepatocyte, both an upregulated A-SMase expression and ceramide content have been related to cell death [89] through activation of death receptors as fatty acid synthase (FAS) and TNFR1 [95]. The induction of necrosis in CCl_4_-treated mice, a fibrosis model widely studied [13,96], has been also correlated with an increase of ceramide content both in serum and liver [97]. Correlated with TNF, previously cited to deplete GSH levels in the cell during NASH, hepatocytes have been described to be more susceptible for cell death in that context [98,99]. Additionally, in mice that lack A-SMase ceramide levels are reduced and lowered TNF-mediated effect is lowered [99]. A-SMase has been also described to activate HSCs which, in the absence of the hepatocyte, promote the development of fibrogenesis through migration and extracellular matrix secretion [100]. 

Not only ceramide has been reported to be upregulated during liver fibrosis and cirrhosis, but also S1P has been pointed out to play a role [94]. It has been characterized that this molecule promotes fibrosis development in cholestatic liver injury by the recruitment of bone-marrow-derived myofibroblasts through S1PR_3_, where an agonist reverts the pathology [101]. The research of the mechanism of action of S1P has been further investigated in another work that characterizes S1P upregulation in human liver fibrosis, as well as the expression of its receptors S1PR_1_ and S1PR_3_ [102], whereas the receptor isoform 2 appears to be downregulated. Indeed, the effect of S1P on myofibroblast migration is also characterized for each receptor, finding a correlation between their expression and their pro-/anti-migratory activity (S1PR_1_ and S1PR_3_ promote migration while S1PR_2_ inhibits) [101]. This work agrees with the study by Liu and colleagues, where the role of S1P in human HSC motility and activation is elucidated [103]. Likewise, S1PR_2_ has been related to regulate and mitigate the fibrotic and regenerative response after liver injury [104]. This could explain the biological effect of S1P in myofibroblasts, where the molecule has been described to exert an anti-proliferative and a pro-survival role [105]. More research is required in order to define the signaling of S1P through each receptor and their regulation.

Despite liver fibrosis being historically considered irreversible, several treatments have proved to improve the pathology [106,107]. Necessarily, the development of a suitable therapy passes through characterizing the main underlying mechanism. Although liver biopsy is the most reliable technique, it presents an elevated cost and risk, resulting in the development of alternative methods, with sphingolipids emerging as a potential diagnosis tool. Changes in metabolic profile have been characterized in fibrosis animal models, such as previously mentioned CCl_4_, with a response to an anti-fibrotic therapy [97,108]. Chang and collaborators have also identified key metabolic changes in a fibrosis rat model, where lactosylceramide levels are increased along the progression of the disease [109]. Such alterations in sphingolipid profile also allow for classification of cirrhosis patients into compensated and non-compensated, relating it with their survival rate [6,110] or associating with another serum marker such as low-density lipoprotein (LDL) levels [111]. Ceramide levels have also been characterized to be altered during necroinflammation in patients with normal alanine aminotransferase (ALT) levels [112], the routine diagnose method in monitoring liver health. 

The research performed in sphingolipid metabolomics has placed these molecules as a potential tool for diagnose cirrhosis patients, stratifying them or determining their survival prognosis. Additionally, targeting S1P-related proteins offers an attractive research line as they reduce the migration and activation of myofibroblasts or HSCs.

## 6. Hepatocellular Carcinoma and Ceramide Metabolism

As aforementioned, NAFLD increases the risk of developing HCC [16,17]. The absence of symptomatology allows diagnosis mostly at advanced stages, leading to a poor prognosis and difficulties during treatment [113,114]. Moreover, recurrence may be enhanced by metabolic reprogramming and different signaling pathways that contribute to the malignant transformation of the HCC and reduce the efficacy of systemic therapies [115]. In order to achieve a more accurate prognosis, an effective diagnostic methods and treatments are required. 

### 6.1. Cancer Development Implies a Reduction of Ceramide Content

Although there is a ceramide increase during NAFLD development at all the stages [76,77,89], ceramide content has been reported to be reduced in HCC leading to a decrease in apoptosis [116] that correlates with the highly proliferative capacity of the disease [117]. Such reduction is caused by the metabolism of ceramide into other bioactive compounds. Despite being mainly characterized in other cancer cell types, the existence of common pathways prompts to expect a similar biological function in liver cancer [118]. 

Ceramide content is reduced through deacetylation and phosphorylation into S1P [119], which plays a proliferative role, especially during DNA damage repair (DDR) mechanisms [120]. Glucosylceramide production, which also reduces ceramide content, acts promoting drug resistance mechanisms during tumor development [121]. Moreover, ceramide phosphorylation into C1P and A-SMase inhibition have been reported to reduce ceramide content [122,123]. Although this ceramide metabolism into C1P has been only reported in macrophages [29], a similar activity of the molecule is expected in KCs from the liver. Furthermore, ceramide transfer protein (CERT), which plays a role during complex sphingolipids synthesis by transferring ceramide from the endoplasmic reticulum to Golgi, has been described to be induced in cancer [124] so related therapies sensitize cells to the treatment [125].

Modulators of ceramide-related enzymes have been emerged as an attractive approach for various cancer types [126]. Anthracyclines have been characterized to increase ceramide content by stimulating CerS triggering apoptosis [127], while Gemcitabine activates A-SMase reaching a similar effect [128]. Cisplatin has been also described to activate A-SMase [129], while the action of S1P-related enzymes (such as S1P lyase [130] or SphK and CerS isoforms [131]) modulates cell sensitivity to the treatment. Focusing on liver-targeting treatments for HCC, Vinblastine has been characterized to activate CerS increasing ceramide content inside the liver [132]. Interestingly, when ceramide is combined with the treatment, an induction of autophagy with the autophagosome maturation is reached, achieving a more powerful antitumor effect [6,118,133]. Another combined treatment with sorafenib and A-SMase promotes cell death relative to the treatment alone [134]. However, despite of sorafenib efficiency being proven, the loss of efficacy, resistance development and side effects [135] make more research required to achieve a better efficacy. 

### 6.2. DDR and S1P as Potential Targets for HCC Therapies

The relationship between NAFLD progression and inflammation has been widely reported, with ROS overproduction and mitochondrial dysfunction leading to the release of pro-inflammatory cytokines as TNF [4,136]. Such ROS overproduction also mediates DNA-involved reactions, causing perturbations that activate DNA damage response (DDR) mechanisms reported to be relevant during HCC development [137]. Despite the exact pro-carcinogenic role of sphingolipids in NASH being poorly understood, some research works have found that S1P upregulates p27 [138], a cell-cycle regulator and a possible prognosis marker as it shows a positive correlation with tumor size [8,139]. Another work from Yin et al., also demonstrate that the alkaline ceramidase ACER3 isoform promotes the proliferation of HCC cells through the formation of S1P [140].

Remarkably, S1P implication has been characterized both in DDR mechanisms and DNA damage [120] while it controls lipid metabolism [8]. Osawa and collaborators have characterized the effect of SphK in hepatoma cell differentiation by promoting the pro-tumor phenotype [141]. In his work they characterize the differentiation effect of inhibiting SphK in Huh-7 cells, an in vitro cell model of human HCC widely used in the study of the disease [142,143]. Particularly, the isoform SphK1 has been related to poor prognosis among cancer patients by having a pro-metastatic role [144,145]. S1P has been pointed out as a key molecule in stimulating invasiveness, angiogenesis, lymphangiogenesis and cell survival against chemotherapy-induced ROS [146,147]. One of S1P-mediated actions is mediated through histone deacetylases (HDACs). The HDAC4 isoform has been described to promote fibrotic liver injury in a prohibitin 1-knockout (*Phb1*^-/-^) animal model which also develops HCC [148,149], suggesting a possible contribution of S1P to the observed effect in tumor development. Taking presented results into account, together with the highly proliferative and angiogenic capacity of HCC [150], S1P emerges as a potential anti-HCC treatment so more research would be interesting to perform.

In summary, most research about sphingolipid contribution to cancer development has not been performed in HCC, so it would be interesting to further characterize the existing turnover from ceramide to S1P in this type of cancer. Compounds targeting this process, as well as the combination with current treatments, seems an attractive approach against the pathology.

## 7. Concluding Remarks

The aim of this work is to point out the relevance of sphingolipids in the development of NAFLD and NAFLD-derived HCC. Presented studies characterize sphingolipids’ (particularly ceramide, SM, and S1P) contribution to NAFLD progression, in each stage of the pathology and its related conditions: MetS, steatosis and NASH, fibrosis/cirrhosis and HCC. Indeed, therapies targeting ceramide- or S1P-related enzymes have been proven to alleviate the condition. Metabolomics and sphingolipid determination in serum also presents an attractive approach for developing diagnostic methods, as these kind of molecules appear to be dysregulated in each pathology.

Ceramide is considered the central molecule in sphingolipids metabolism and its modulation plays a key role in the development of several pathologies. In an early steatosis condition, ceramide accumulation could be only a direct consequence of an imbalance, mainly determined by a perturbation in hepatic lipid content, as it is not exerting its biological pro-apoptotic function. This might be due to the ACSL5-DGAT2-CerS complex, which would somehow “buffering” ceramide effect by converting it into acylceramide. In more advanced stages, the complex could not afford such ceramide overproduction so ceramide would be mainly in its free form, promoting hepatocyte death and its metabolism into S1P. Meanwhile, S1P activates HSCs contributing to fibrosis development by ECM secretion and replacement of death hepatocytes. Finally, at the last stage of chronic disease such as HCC, an activation of the enzymes that reduce ceramide content in the cell is stimulated. This ceramide reduction prevents the cells from the anti-proliferative effect, so that this would favor tumor growth. Therein, S1P is the most abundant sphingolipid in the liver and plays a role in proliferation, invasiveness and angiogenesis capacity of the tumor (See graphical abstract).
